# Reciprocity in Medical Licensure1From the New York Medical Journal, October 13, 1906.

**Published:** 1907-01

**Authors:** Albert Vander Veer

**Affiliations:** Albany, N. Y.; Regent of the University of the State of New York


### BUFFALO MEDICAL JOURNAL.

VOL. LXII. JANUARY 1907. No. 6

INDEX THROUGH

### ORIGINAL COMMUNICATIONS.

### Reciprocity in Medical Licensure.[1]

By ALBERT VANDER VEER, M. D., Albany, N. Y.
Regent of the University of the State of New York.

AS a result of conferences between the States of New Jersey, Michigan, and Ohio, formal agreements for reciprocity in medical licensure have been entered into between the States of New York and New Jersy, Michigan, and Ohio, during the school year just closed.

The basis upon which reciprocity obtains between these states is a license earned *on examination* in either one of them. The candidate for endorsement of a medical license must present credentials from the officials of the state board of medical examiners which licensed him, showing that at the time of such application he is a reputable practitioner. Provision is made for the inspection of the qualifications of an applicant either personally or professionally when there are reasonable doubts of his qualifications, and an applicant presenting a license issued prior to the establishment of reciprocity may be required to submit the original papers on which the license was granted or certified copies thereof. Only an original state license can be endorsed.

The preliminary education required for admission to the medical schools must be the same in each state, and the certification of the education department of the state as to the standards maintained by secondary schools will be accepted by the education department of the other states. The standards to be required of secondary schools without those states must be fully equivalent to those required within the states, and such standards shall be determined in joint conference between the education departments of the states, the registered list of New York State remaining in force till a joint list becomes operative.

The unit of value in measuring or estimating the preliminary qualifications is the count which is of universal use in New York state and is in accord with the academic syllabus for secondary

1. From the New York Medical Journal, October 13, 1906.

schools. The recognized medical schools registered as maintaining the required standards are those of the states in which the applicant seeks endorsement. The standard of registration of the board of regents of the University of the State of New York and their list of registered medical schools in group 1 are adopted, each state reserving the right of submitting evidence with reference to any institution, either for removing it from or for placing it on the approved list.

Full faith and credit are given by the board of each state to the examinations held by the boards of the other states. The applications for license under this agreement must be endorsed in the representative states by the president and secretary of the board of examiners and by the commissioner of education. The agreement has been signed by the representatives of the state boards and the education departments, and remains in force until rescinded by formal action.

From the report of an American Consul in Austria, Hugo Donzelmann, Prague, March, 1898, the following statement of the principles of the first laws promulgated in Europe with reference to the practice of medicine and pharmacy is condensed. Their principle is still apparent in all later laws, namely, the public good, and this principle is the underlying principle in the growth of laws affecting admission to the practice of the learned professions in the United States.

The first decree of Frederick II., in 1224, can be said to have been the fundamental constitution of all existing laws in Europe, the same having been amended and improved upon from time to time, but always bearing in mind that the practice of medicine and pharmacy was to be under the special care and supervision of the government in order to protect its people against imposition. In that year he established by a decree the first college for the education of physicians, at Naples, and promulgated the first laws governing the practice of medicine within his domain, viz., that no person should be admitted to the practice of medicine who had not passed his examination before the Collegio Medico de Napoli; that after having received his diploma from said college it became necessary for the person to enter into active practice with a regular practising physician for the period of one year as assistant; and that an oath had to be taken by the person whereby he promised to follow and live up to the laws of the country respecting the practice and sale of medicine and whereby he bound himself to attend to the sick, to accept only a reasonable fee from all who were able to pay, and to treat the poor and impecunious free and without charge.

It would be interesting in this connection to trace the development of professional laws in the United States, but this is foreign to the purpose of this article, and I have time only to refer to the series of bulletins published by the regents, entitled *Professional Education in the United States, Medicine.* The New York statute establishing these standards whereon reciprocity may be entered into with other states, reads as follows:—

"Applicants examined and licensed by other state examining boards registered by the regents as maintaining standards not lower than those provided by this article, may, without further examination, on payment of $10 to the regents, and on submitting such evidence as they may require, receive from them the endorsement of their license or diploma, conferring all rights and privileges of a regents' license issued after examination."

In determining whether standards are lower than those provided by the statute, four distinct lines of statutory requirements are studied: (1) the preliminary education required for admission to the professional school; (2) the professional training required for graduation; (3) the licensing test required by an independent examining board; and (4) registry in offices of record.

The present preliminary education of New York state required for admission to registered medical schools and the medical licensing examination, is evidence of four full years of secondary education subsequent to eight years of elementary or the equivalent in higher institutions. The evidence of this preliminary education is the medical student certificate, in the determination of which certain specific requirements are exacted. By reason of the unification of the various educational forces of the state a harmonious and complete determination of the various conditions surrounding the problem of admission to higher institutions is possible. For example, the more than 650 high schools and 150 academies are chartered or admitted to membership in the university under uniform requirements. The courses of study in these secondary institutions are based on eight full years of elementary instruction brought into harmony with the secondary work through a centralized administration. The subjects and their treatment in the secondary schools are outlined and determined on expert judgment of the teaching body through its representatives and the appropriate authorities in the education department, which finds expression in the academic syllabus in force for a syllabus period of five years. The work of these secondary schools is tested and kept up to grade by a system of examinations which not only provides for this purpose, but renders possible the determination of the character of work done by students unable to take advantage of the schools.

The registration of the secondary schools based on inspection and examination tests provides for a series of credentials the most accurate and clearly determined by any administrative authority.

By reference to the full agreement in effect August, 1906, between the states of New York and Ohio, Section 5 (see page xx) it will be seen that the preliminary education required for admission shall be the same for both states, and that the certification of the education department of either state shall be accepted by the education department of the other. The effect of this agreement, as shown by experience with New Jersey, is to develop and harmonize the requirements for admission to the other professions, so that in the near future there will be reciprocal relations, not only in medical licensure, but in all other lines of affiliated relations, with a number of the strongest states of the Union.

The professional requirement for the registration of medical schools and for admission to the medical licensing examination is the study of medicine not less than four full school years or at least nine months each, including four satisfactory courses of at least six months each in four different calendar years in a medical school registered as maintaining at the time a satisfactory standard. The registration of the medical schools has reference to the professional educational requirement and, for all schools registered or accredited July 1, 1906, and subsequent thereto, to the general preliminary educational requirement also that must be met by candidates for admission to New York medical schools. Prior to that date the registration of the professional requirements of medical schools was determined independently of the general preliminary education required for admission to the same. A marked advance has come from the accuracy and careful consideration of the problems involved in registration. When registration required the meeting of the full professional law, and no crediting was possible, students in lower institutions desiring to migrate to superior found it impossible to do so, for the authorities of the lower institutions would not apply formally for registration when they feared the loss of students, but by the system of registering in two groups students can receive credit for such work in medicine as can be accorded them, and relieved from repeating the same in registered institutions.

The formal licensing examination is but one of the three essentials to a license to practise medicine in the state. The first essential is based on the principle that the student had completed his preparatory work prior to entrance on his professional, with the possibility of his being conditioned in not more than one year of the preliminary training, *which must be worked off before beginning the second course counted toward the degree.* This latter provision is a source of considerable friction in the administration of the law and is but little understood by the general public. Its wisdom, however, is plainly apparent in the development of this work.

*The second essential,* a degree from a registered professional school, is a marked step in advance of the requirements in foreign countries, as it is well known that very few of the medical practitioners of Germany and Russia complete the requirements for a degree, the state's examination being regarded as even a higher qualification. Moreover, the determination of this requirement exacts careful attention to certain details. The four full years of medicine necessitates at least forty-five months of professional training; full years of medical training precludes an allowance to graduates of other cognate professional schools, such as dentistry, veterinary medicine, pharmacy, osteopathy, and the like, but the accuracy of this registration and the determination of secondary units of measure are leading to the possibility of recognition of work completed in kindred professional schools. It needs no argument to convince one that the graduate of a medical school having a four years' curriculum possesses training that can be accepted as a portion of the admission requirements to other professional schools. It is also apparent that if accurate definitions become available, work in professional schools of one character may be recognized in lieu of the same work in other schools of kindred nature. For example, general chemistry, the foundation for the special chemistry in a medical school, should receive credit for the same general preparation for admission to the special chemistry of a pharmacy school.

*The third essential* is the formal written licensing examination, which is based on the former essentials and is the final act in the government's special care for the public good. The question of the use of English does not enter into the revision of the applicant's paper, although he is obliged to use the English language in his answers, for his mental training was determined by the first essential requirement. No effort is made by the formal written test to determine the clinical experience of an applicant, such requirement having been met under the second essential. The final test—the licensing examination—determines the competency of the candidate applying for admission to the *general* practice of medicine in the state, in order to protect the people against imposition.

The importance of these agreements can hardly be overestimated, and to show the marked advance of this step I quote from Bulletin 8, *Professional Education in the United States,* entitled, *Medicine,* issued January, 1900:—

"A uniform standard for admission throughout the United States is impracticable at present, owing to varying conditions as to density of population, educational advantages, and general development. Weak states cannot maintain the standards maintained elsewhere, and strong states cannot afford to lower their standards. The present need of less multiplication of standards, however, is most important. Instead of a separate standard for almost each political division, two or at most three standards should answer for all. In the first group should come the strongest states, and the standards maintained by these states would act as a stimulus to weaker political divisions."

From a careful consideration of the *synopsis* published in *Handbook* 9 of the education department, it plainly appears that reciprocity can become effective in the near future between a group of states extending from the Atlantic to the Pacific.

It can be said without fear of successful contradiction that to-day the New York statutory requirement for admission to the practice of medicine is clearly equal to the highest required by any state or government in the civilized world. While it may be said that the requirements for state's examination in Prussia exacts higher preliminary and professional training, a recent communication from the Prussian authorities asserts that they are not obligatory on applicants for admission to the practice of medicine in Germany. While the requirements in Austria are plainly superior to those of other European countries, with the possible exception of France and Switzerland, the problem of notification has complicated the situation and the strained relations between Austria and Hungary have led to modifications that obscure the view. It is worthy of comment that the highest requirements for admission to the practice of medicine are found, not under monarchial governments, but under republican forms.

As to the future, the study and attention given to the problems involved in items reported above bid fair to receive final solution in the near future, and we look to the development in the present decade of preliminary requirements for admission to professional schools that shall equal the standards of Germany; professional training in higher institutions the full equivalent of that of Austria, Hungary, or Italy; the combination of university courses whereby the work required for degrees in arts and sciences, in medicine and like departments, shall be so coordinated as to require the least repetition and accord the highest recognition to all scientific work in these departments, which form the basis for the special work of the schools. An earnest effort is being made to so correlate the courses of instruction in elementary, secondary, and higher institutions of the state as to save from one to two years' time in the life of the student attaining a combined baccalaureate and medical degree.

In closing, let it be said that New York State has little desire to exploit its standards and less to insist on other states being satisfied with these minimum requirements.

28 EAGLE STREET.

